# Bcl-2抑制剂维奈克拉联合用药治疗IDH1/2突变阳性急性髓系白血病的疗效及安全性分析

**DOI:** 10.3760/cma.j.issn.0253-2727.2022.08.014

**Published:** 2022-08

**Authors:** 馨 王, 协炳 鲍, 剑 张, 宝全 宋, 梦云 郦, 珺丹 解, 宏杰 沈, 德沛 吴, 惠英 仇

**Affiliations:** 国家血液系统疾病临床医学研究中心、江苏省血液研究所、苏州大学附属第一医院血液科，苏州 215006 National Clinical Research Center for Hematologic Diseases, Jiangsu Institute of Hematology, Department of Hematology, The First Affiliated Hospital of Soochow University, Suzhou 215006, China

异柠檬酸脱氢酶（IDH1/2）突变是成人急性髓系白血病（AML）中常见的基因突变，IDH1及IDH2基因在成人AML中的突变率分别为5.5％～10.4％及8.6％～17.7％[1]。伴有IDH1和IDH2的患者常预后不良[2]，在国内对特异的靶向药物IDH1抑制剂ivosidenib和IDH2抑制剂enasidenib可及性不高的情况下，我们采用Bcl-2抑制剂维奈克拉（VEN）治疗30例伴IDH1/2基因突变的AML患者，观察其疗效及不良反应，现报道如下。

## 病例与方法

1. 病例资料：回顾性分析2018年8月至2021年8月在苏州大学附属第一医院确诊的121例IDH1/2突变阳性AML患者的临床资料，共计30例患者接受了VEN为主的联合治疗，其中29例联合去甲基化药物，1例同时联合了CAG方案预激化疗。余91例均接受未联合VEN的治疗方案。所有患者经骨髓细胞形态学、白血病免疫分型、细胞遗传学、分子生物学等检查确诊。

2. 治疗方案：VEN 100 mg第1天，200 mg第2天，400 mg第3～28天，口服，用药期间根据骨髓抑制情况及耐受情况调整剂量。2例患者因开始用药时即存在粒细胞缺乏（粒缺，中性粒细胞绝对计数<0.5×10^9^/L），VEN最大剂量调整为100 mg/d，粒缺同时加用伏立康唑预防真菌感染。后续用药过程中有28例患者因出现粒缺下调VEN剂量至100 mg/d，同时加用伏立康唑预防真菌感染。重度粒缺（<0.2×10^9^/L）合并重度感染时暂停口服VEN。用药期间监测血常规，骨髓抑制期HGB<60 g/L或出现明显贫血症状时输注悬浮红细胞，PLT<20×10^9^/L或有明显出血倾向时输注血小板。VEN用药第14天复查骨髓，若形态学完全缓解（原始细胞<5％）加用G-CSF，直至脱离粒缺（中性粒细胞绝对计数>0.5×10^9^/L）且脱离血小板输注，并将VEN加量至200 mg/d或400 mg/d，同时停用伏立康唑。

3. 疗效和不良反应评估：参照欧洲白血病网（ELN）2017版关于成人AML诊治建议[3]判定疗效，包括完全缓解（CR）、CR伴血液学不完全恢复（CRi）、形态学无白血病状态（MLFS）、部分缓解（PR）和未缓解（NR）。总体反应率（ORR）定义为患者在治疗后达到CR、CRi、PR、MLFS的比例。治疗期间不良反应的评价依据常见不良事件评价标准（CTCAE）5.0版。总生存（OS）时间定义为从进入VEN联合用药治疗起至任何原因所致死亡或末次随访的时间。无事件生存（EFS）时间定义为从进入VEN联合用药治疗起至疾病进展、复发、任何原因所致死亡或末次随访的时间。

4. 随访：采用门诊复查、电话或短信方式随访，随访截止日期为2021年9月12日。

5. 统计学处理：应用SPSS 26.0软件进行统计分析。患者特征通过连续变量的中位数（范围）和分类变量的频数（百分比）进行描述。分类变量的比较采用卡方检验或Fisher确切概率法。生存分析采用Kaplan-Meier法绘制生存曲线和Log-rank检验进行单因素分析。双侧检验*P*<0.05判定为差异有统计学意义。

## 结果

1. 一般资料：30例接受VEN联合治疗的患者中，初治16例，难治5例，化疗后复发8例，移植后复发1例。男14例，女16例，中位年龄55（26～78）岁，用药前骨髓中位原始细胞比例53.0％（5.5％～91.0％）。30例患者中位基因突变数目4（2～8）个，IDH中位突变率为38.5％（2.0％～49.1％），IDH1中位突变率高于IDH2中位突变率（43.0％对34.0％，*P*＝0.162）。高频共突变主要为DNMT3A突变（14例）、RUNX1突变（7例）和FLT3-ITD突变（6例），且与DNMT3A共突变更多见于IDH2组。依据ELN 2017版AML遗传学风险分层，预后良好组7例，预后中等组11例，预后不良组12例。91例接受未联合VEN治疗组患者均为初治，两组患者的临床特征比较详见表1。

**表1:** 联合维奈克拉（VEN）组与未联合组IDH突变阳性急性髓系白血病（AML）患者的临床特征

临床特征	联合VEN组（30例）	未联合VEN组（91例）	*P*值
性别［例（％）］			0.271
男	14（46.7）	53(58.2)	
女	16（53.3）	38(41.8)	
年龄［岁，*M*（范围）］	55（26～78）	52（17～74）	0.368
初诊WBC［×10^9^/L，*M*（范围）］	3.8（1.0～189.0）	5.9（0.4～262.2）	0.577
初诊HGB［g/L，*M*（范围）］	93（39～124）	85（27～117）	0.519
初诊PLT［×10^9^/L，*M（*范围）］	84（13～683）	52（4～804）	0.018
初诊骨髓原始幼稚细胞比例［％，*M*（范围）］	61.5（15.5～91.0）	61.6（8.0～93.0）	0.780
核型分析［例（％）］			
正常核型	21（70.0）	62（68.1）	0.849
t（8;21）	2（6.7）	4（4.4）	0.621
复杂核型	2（6.7）	2（2.2）	0.237
其他不良预后核型	1（3.3）	8（8.8）	0.325
其他中危核型	4（13.3）	15（16.5）	0.682
IDH突变类型［例（％）］			
IDH1	11（36.7）	37（40.7）	0.830
IDH2	18（60.0）	52（57.1）	0.834
IDH1、IDH2共突变	1（3.3）	2（2.2）	1.000
遗传风险分层［例（％）］			
良好	7（23.3）	32（35.1）	0.231
中等	11（36.7）	29（31.9）	0.629
不良	12（40.0）	30（33.0）	0.485

2. 疗效评价：1个疗程VEN联合用药治疗后评估，30例患者中CR 22例（73.3％），CRi 2例（6.7％），PR 1例（3.3％），NR 5例（16.7％），ORR为83.3％（25/30）。16例初治患者CR 15例（15/16），PR 1例（1/16）；9例复发患者CR 5例（5/9），NR 4例（4/9），需要注意的是，1例移植后复发患者在VEN治疗后再次获得CR；5例难治患者CR 4例（4/5），NR 1例（1/5）。初治患者的ORR高于复发/难治患者（100％对64.3％），差异有统计学意义（*P*＝0.014）。IDH1突变患者的ORR与IDH2突变患者相近（90.9％对83.3％，*P*>0.05）。进一步分析发现，IDH突变不伴FLT3-ITD突变组的ORR高于IDH、FLT3-ITD共突变组（87.5％对66.7％），差异无统计学意义（*P*＝0.186）；同样的结论也在IDH不伴RUNX1突变组与IDH、RUNX1共突变组得出（87.0％对71.4％，*P*＝0.565）。而IDH、DNMT3A共突变组的ORR与不伴DNMT3A突变组的ORR率相近（78.6％对87.5％，*P*＝0.642）。此外我们发现预后良好组、预后中等组与预后不良组的ORR率差异无统计学意义（分别为71.4％、100.0％、75.0％，*P*>0.05）（图1）。

**图1:**
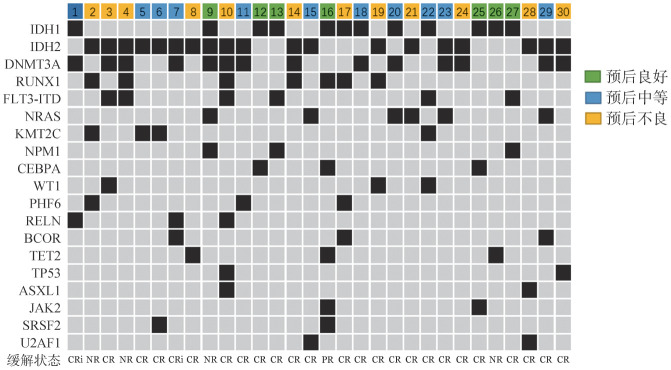
30例伴IDH突变急性髓系白血病（AML）患者高频共突变情况及维奈托克联合用药治疗情况

91例初治未联合VEN患者经1个疗程诱导治疗后评估，CR 50例（54.9％），CRi 6例（6.6％），PR 13例（14.3％），NR 22例（24.2％），ORR为75.8％（69/91）。初治IDH1/2突变阳性AML患者中联合VEN组的ORR显著高于未联合组（100％对75.8％，*P*＝0.039）。91例患者中22例为复发/难治患者，其中16例接受未联合VEN的再诱导化疗，治疗后评估CR 6例（37.5％），NR 10例（62.5％）。复发/难治IDH1/2突变阳性AML患者中联合VEN组的CR率高于未联合组（64.3％对37.5％），差异无统计学意义（*P*＝0.272）。

3. 随访和预后：中位随访7.5（1.0～29.0）个月，30例接受VEN联合治疗患者的中位OS时间为21.5个月。联合VEN组患者的1年EFS率、1年OS率和未联合VEN组患者相比，差异均无统计学意义（58.1％对70.0％，*P*＝0.113；63.0％对75.4％，*P*＝0.365）。30例联合VEN患者中，1例PR患者在开始治疗第36天死于败血症合并肺泡出血，其余29例均接受后续治疗。共计8例患者后续接受异基因造血干细胞移植（allo-HSCT），6例CR后桥接移植，2例非CR状态下行挽救性移植，均使用改良BUCY方案预处理。2例行挽救性移植患者在移植后第30天评估骨髓状态时均获得CR。截至随访结束，8例后续行allo-HSCT患者中1例复发（缓解时间为126 d），CR后桥接移植患者未达到中位OS时间，挽救性移植患者中位OS时间为4.0个月。21例患者后续仅接受化疗，截至随访结束，共计6例复发，3例本病进展。剔除1例IDH1及IDH2共突变患者，IDH1突变阳性组和IDH2突变阳性组的OS和EFS差异均无统计学意义。VEN治疗后获ORR组未达到中位OS时间，而NR患者仅为4.0个月，与NR的患者相比，获得ORR的患者的1年EFS率（49.1％对0，*P*＝0.003）、1年OS率明显提高（68.8％对0，*P*＝0.001）。

4. 不良反应：截至2021年9月12日，30例患者中20例（66.7％）存活，10例（33.3％）死亡。死亡原因包括复发4例、本病进展2例、allo-HSCT后感染2例、化疗后败血症合并肺泡出血1例、脑出血1例。所有患者均发生3～4级血液学不良反应，包括骨髓造血受抑、白细胞减少、中性粒细胞减少、血小板减少，治疗过程中13例（43.3％）患者出现粒缺期发热。30例患者脱离粒缺中位时间17（7～42）d，中位HGB最低值为57（40～103）g/L，中位PLT最低值为16（4～131）×10^9^/L，治疗期间红细胞和血小板平均输注量分别为4单位和3.5单位。最常见的非血液学不良反应为感染（43.3％），其中肺部感染多见（23.3％），其余还包括1～2级恶心呕吐（20.0％）、1～2级肝功能不全（6.67％）。1例患者败血症合并肺泡出血，经抗感染、止血治疗无效后死亡，其余患者均经积极对症治疗后好转。IDH1突变患者的感染发生率高于IDH2突变患者（63.6％对35.3％），差异无统计学意义（*P*＝0.246）。预后良好组、预后中等组与预后不良组感染发生率差异无统计学意义（*P*>0.05）。

## 讨论

在AML患者中，IDH1/2基因突变是杂合错义突变，突变的IDH1和IDH2酶催化α-酮戊二酸（α-KG）还原转化为肿瘤代谢物2-羟基戊二酸（2-HG）[4]–[5]。2-HG在白血病发生过程中参与多种机制，包括诱导组蛋白修饰[6]–[7]及干扰造血细胞的分化[8]–[9]等。伴IDH突变AML常具有OS率低、无病生存期短及复发率高的特点[2]，且常见于老年患者，随年龄的增长频率增加[10]–[11]。因此对于IDH突变的AML患者，尤其是其中不适合强化疗患者（如年龄大、脏器功能差）或者某些复发难治（R/R）患者，选择何种化疗方案从而提高其生存时间、减少化疗相关并发症一直是研究的重点。VEN是一种口服小分子BCL-2抑制剂，具有广泛抗肿瘤活性，但AML患者敏感程度存在很大差异。而Chan等[12]通过大规模RNA干扰（RNAi）筛选确定了BCL-2为IDH突变的合成致死基因，并且无论在体外和体内模型中，具有IDH突变的AML细胞比野生型细胞对VEN更敏感。表明IDH突变状态是影响BCL-2抑制剂敏感性的因素之一，这也有助于识别对BCL-2抑制剂可能有反应的患者。

DiNardo等[13]研究发现对于IDH突变的AML患者，联合VEN组患者的1年OS率显著高于未联合VEN组（66.8％对35.7％）。在我们的研究中，30例患者的1年OS率为63.0％，与报道相似，但联合VEN组的1年OS率与未联合VEN组接近。Pollyea等[14]报道具有IDH突变的初治AML患者经VEN联合治疗后的CR/CRi率达78.5％。我们的研究表明，初治患者极大获益于VEN联合方案，其CR/CRi率可达93.8％，且应答率也显著高于未联合VEN组（100％对75.8％，*P*＝0.039）。同样地，R/R AML患者也可获益于VEN联合方案，R/R AML患者中联合VEN组的CR率高于未联合VEN组（64.3对37.5％，*P*＝0.272）。我们也发现，联合VEN组的初治AML的ORR显著高于R/R AML（100％对 64.3％，*P*＝0.014），这可能与R/R患者原发或继发耐药等相关。此外，不伴FLT3-ITD突变组较IDH、FLT3-ITD共突变组显示出对VEN更好的应答率，不伴RUNX1突变组较IDH、RUNX1共突变组也显示出对VEN更好的应答率。因此我们可得出：对于伴IDH突变AML，难治、FLT3-ITD共突变、RUNX1共突变可能是影响VEN联合用药疗效的预测因子。但这需要更多的临床数据验证。主要不良事件方面，研究发现联合VEN组患者治疗时均出现了3～4级血液学不良反应，85％的患者发生感染[13]。在我们的研究中，血液系统不良反应与报道一致，而感染的发生率明显降低（43.3％）。尽管伴IDH突变的AML对基于VEN的疗法反应特别好，在持续缓解和生存方面均具有明显获益。但是，VEN在应用中存在原发及获得性耐药，相关机制包括烟酰胺代谢升高、单核细胞亚克隆、TP53凋亡网络等[15]–[17]。因此如何进一步发挥其疗效，需要尝试更多的组合方案。

综上所述，本研究回顾性分析了30例伴IDH1/2基因突变的AML患者使用Bcl-2抑制剂VEN联合治疗的疗效及安全性，结果显示VEN联合用药缓解率高、OS及EFS时间长，初治AML及R/R AML均可显著获益，并且不良事件少，可作为IDH1/2基因突变阳性AML的有效治疗选择之一。但由于本研究是单中心回顾性病例研究，病历资料少，所得结论还有待更多样本量、多中心的临床研究进一步确证。
